# Advances in therapeutic lesioning in functional neurosurgery

**DOI:** 10.3171/2024.7.FOCVID23220

**Published:** 2024-10-01

**Authors:** Andres M. Lozano, Ellen L. Air, Gordon H. Baltuch, Francisco A. Ponce

**Affiliations:** 1 Division of Neurosurgery, University of Toronto, Ontario, Canada;; 2 Department of Neurosurgery, Henry Ford Health, Detroit, Michigan;; 3 Department of Neurological Surgery, Columbia University, New York, New York; and; 4 Department of Neurosurgery, Barrow Neurological Institute, Phoenix, Arizona

Functional neurosurgical procedures, including deep brain stimulation, are being used in the context of movement disorders, pain, epilepsy, and psychiatric disturbances. Despite the track record of safety and proven efficacy of such procedures, the use of deep brain stimulation has been slow to permeate the patient population. The reasons for this are multifactorial but in part relate to the patient’s reluctance to undergo an invasive neurosurgical procedure. This is where the possibility of noninvasive therapies, specifically focused ultrasound, may play an important role. Because focused ultrasound does not rely on obtaining direct measures of neurophysiological activity in the brain, the anatomy of the target structures and adjacent tracks becomes of utmost importance. In this issue of *Neurosurgical Focus: Video*, we concentrate on therapeutic lesioning in the central nervous system to treat movement disorders, using both traditional open methods with radiofrequency lesioning and noninvasive methods, specifically focused ultrasound. Fundamental to every one of these procedures is a clear understanding of the anatomy and function of the brain target structures and the adjacent neural tracks. In performing lesion surgery without the benefit of precise physiological mapping, the technical nuances of MR-guided focused ultrasound procedures require meticulous attention to detail to make the lesions in the right place and to ensure that the lesions do not encroach on adjacent structures. The advent of diffusion tensor MRI is offering an unprecedented appreciation and details of white matter pathways that are important in functional neurosurgery interventions. In this issue, we discuss some of the fundamental neuroanatomical considerations in these procedures. In functional neurosurgical procedures for movement disorders, the authors go through the step-by-step technological aims of these procedures and cover some of the practical issues that underlie starting these types of therapies. These issues are important for those who are new to performing functional neurosurgical procedures, as well as for those developing them, by the addition of noninvasive methods, including focused ultrasound.

## Disclosures

Dr. Lozano reported consulting fees from Abbott, Boston Scientific, Insightec, and Medtronic; and personal fees (scientific director) from Functional Neuromodulation. Dr. Air reported research fees from Medtronic; and personal fees from Enspire DBS, Functional Neuromodulation, Stryker, and Setpoint.

